# The complete mitochondrial genome of the *Chilotilapia rhoadesii*

**DOI:** 10.1080/23802359.2017.1334522

**Published:** 2017-05-31

**Authors:** Xiangru Wen, Cailin Wang, Man Li, Haiyan Wang, Xu Zhou

**Affiliations:** School of Basic Education Sciences, Xuzhou Medical University, Xuzhou, China

**Keywords:** Mitochondrial genome, *Chilotilapia rhoadesii*, phylogenic relationship

## Abstract

In this study, we firstly reported the complete mitochondrial genome of *Chilotilapia rhoadesii*. The whole mitochondrial genome is 16,580 bp in length, including 2 ribosomal RNA genes, 22 transfer RNA genes and 13 protein-coding genes. Its GC content is 45.98%, similar to Alticorpus geoffreyi (45.82%). We also analyzed the complete mitochondrial genome of *C. rhoadesii* and its phylogenic relationship with other 14 related species, which would facilitate our understanding of the evolution of Cichlidae mitochondrial genome.

*Chilotilapia rhoadesii* is a member of Cichlidae family. In this study, we firstly reported the complete mitochondrial genome of *C. rhoadesii*, which would help understand the phylogenic relationship of the Cichlidae family.

Here, we assembled the complete mitochondrial genome of *C. rhoadesii* (Genbank accession: MF033353) based on the raw data of the whole genome of a *C. rhoadesii* (SRA: ERP002088). The sample was collected in the Southeast Arm of Lake Malawi and sequenced by the Wellcome Trust Sanger Institute (SC) in their Cichlid diversity sequencing WTMGM student project. We used SOAPaligner/soap2 (V2.21) (Li et al. 2009) to map all the raw reads to the reference mitochondrial genome, *Alticorpus geoffreyi* (Genbank accession: NC_028033) (Qi et al. 2016). To get the complete mitochondrial genome of *C. rhoadesii*, we assembled these reads which could map to the reference genome by SPAdes3 (V3.1.0) (Bankevich et al. 2012). Moreover, DOGMA (Wyman et al. 2004) and tRNAscan-SE 2.0(Lowe & Eddy 1997) were used to annotate this mitochondrial genome.

The mitochondrial genome of *C. rhoadesii* is 16,580bp in length, including 2 ribosomal RNA genes (rRNA), 22 transfer RNA genes (tRNA) and 13 protein-coding genes (PCGs). Its GC content is 45.98% (27.39% A, 26.62%T, 30.08% C, 15.90% G), similar to A. geoffreyi (45.82%) from the same family, Cichlidae. The lengths of 22 tRNA genes range from 67 bp (tRNA^Cys^ and tRNA^Ser^) to 74 bp (tRNA^Leu^ and tRNA^Lys^), whereas 16S rRNA is 1675bp and 12S rRNA is 941bp. All PCGs in *C. rhoadesii* are started with ATG and stopped with TAN or AGA, except for COX1 started with GTG.

Furthermore, we used MEGA7 (V7.0.25) (Kumar et al. 2016) to construct the phylogenetic tree on the complete mitochondrial genomes of *C. rhoadesii* and other 14 related species (Figure 1).

**Figure 1.**
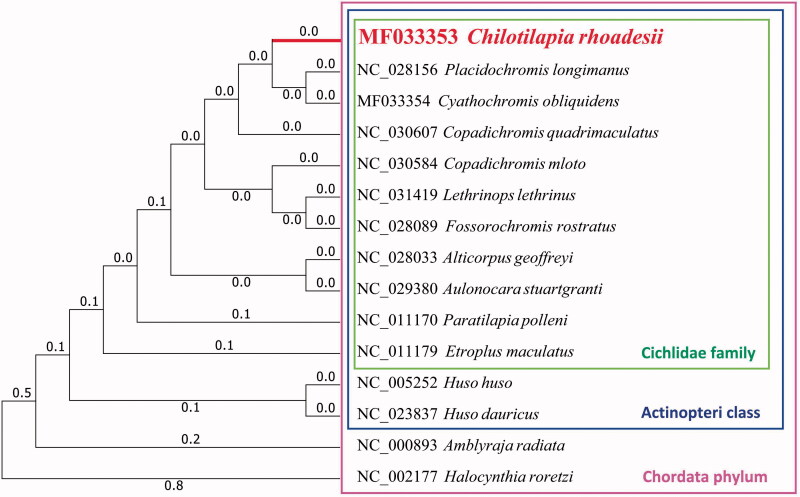
Maximum likelihood tree of complete mitochondrial genome of *C. rhoadesii* and 14 other closely species, which have their Genbank accession numbers in front.
